# Supplementary figures and images for: NOMO-1 cells expressing an NF-κB luciferase reporter gene facilitate a simple, rapid monocyte activation test that can detect a wide range of pyrogens
Source: PLoS One. 2025 Jun 20;20(6):e0326408. doi: 10.1371/journal.pone.0326408 (PMC12180646; doi:10.1371/journal.pone.0326408)

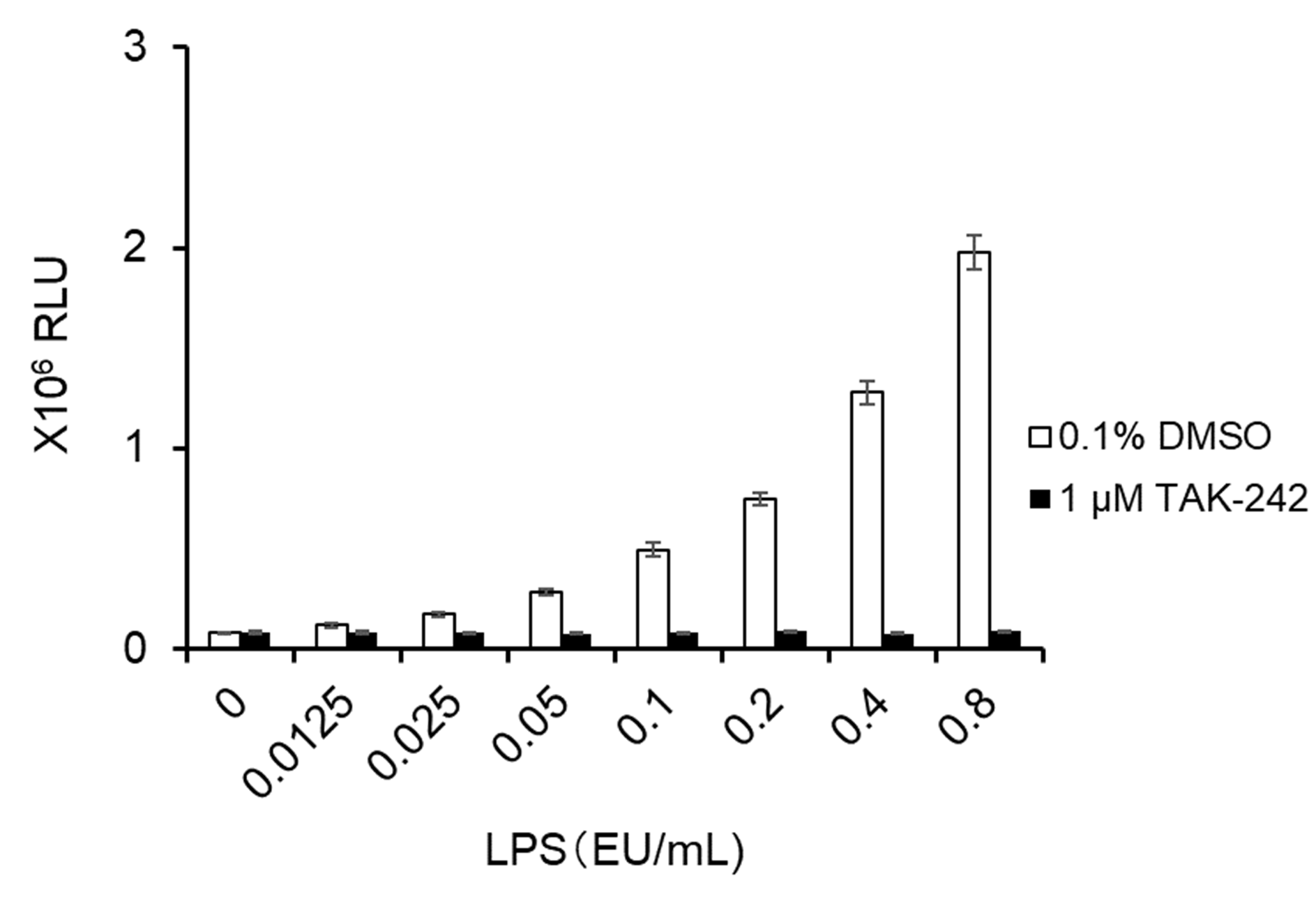

Supplement: S1 Fig — TAK-242, a Toll-like receptor (TLR) 4 inhibitor, was added (final concentration, 1 µM) to the mixture of lipopolysaccharide (LPS) standard and the stable reporter cells and incubated for 3 hours. Bar (SD): n = 4 wells. (TIF) [file pone.0326408.s001.tif]

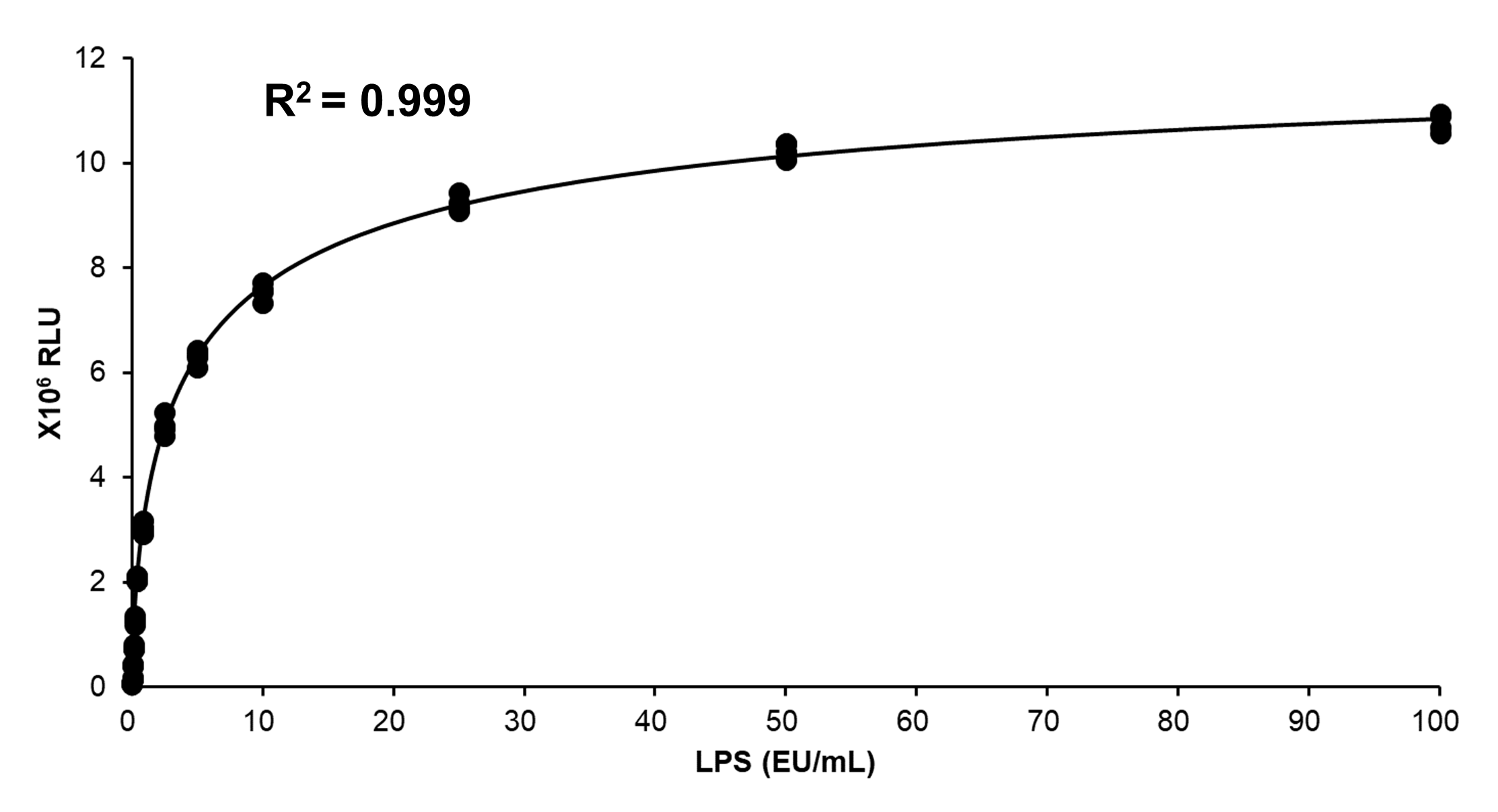

Supplement: S2 Fig — A four-parameter logistic standard curve was created on the basis of the response of the NF-κB reporter gene to lipopolysaccharide (LPS) treatment. For each well, a cell suspension of 5 × 10^4^ cells/50 µL was mixed with 50 µL of LPS standard (0.0125, 0.025, 0.05, 0.1, 0.2, 0.4, 0.8, 2.5, 5, 10, 25, 50, and 100 EU/mL) and incubated for 3 hours. Each LPS concentration was measured in quadruplicate. (TIF) [file pone.0326408.s002.tif]

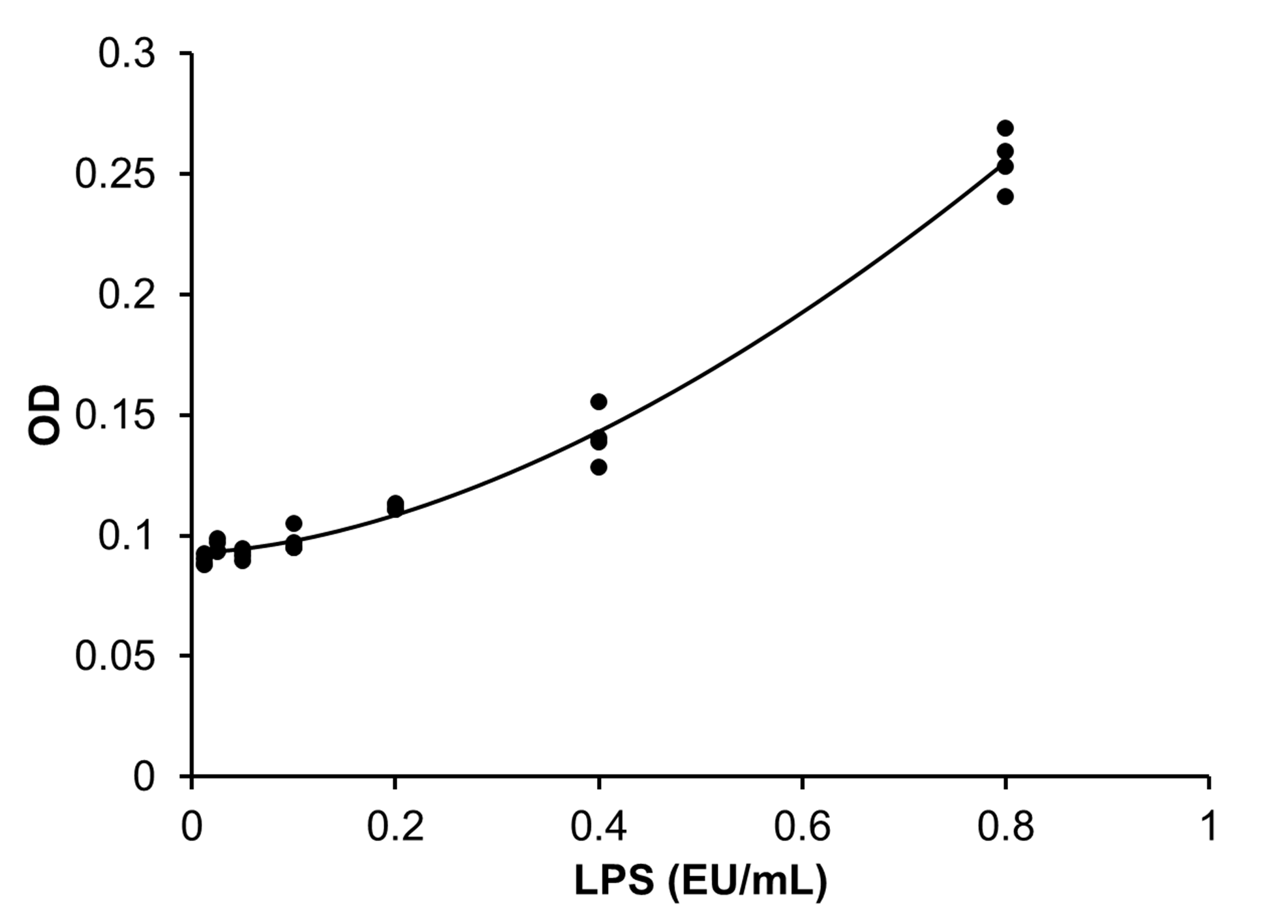

Supplement: S3 Fig — A cell suspension of 1 × 10^5^ cells/100 µL was mixed with 100 µL of lipopolysaccharide (LPS) standard (0.0125, 0.025, 0.05, 0.1, 0.2, 0.4, and 0.8 EU/mL) in each well and incubated for 22 hours. After incubation, 50 µL of the culture supernatant was used for an enzyme-linked immunosorbent assay. Each LPS concentration was measured in quadruplicate. (TIF) [file pone.0326408.s003.tif]

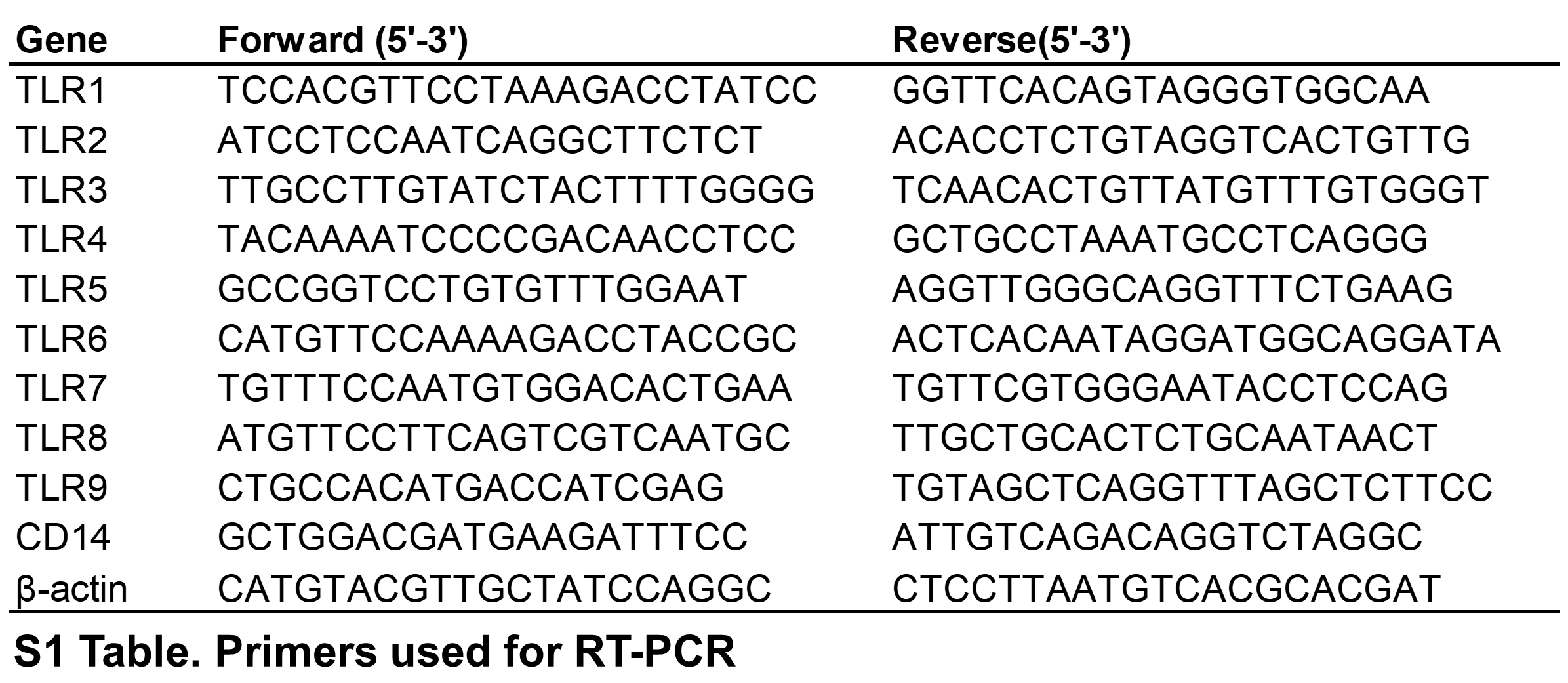

Supplement: S1 Table — (TIF) [file pone.0326408.s004.tif]
