# Supplementary material for: NOMO-1 cells expressing an NF-κB luciferase reporter gene facilitate a simple, rapid monocyte activation test that can detect a wide range of pyrogens
Source: PLoS One. 2025 Jun 20;20(6):e0326408. doi: 10.1371/journal.pone.0326408 (PMC12180646; doi:10.1371/journal.pone.0326408)
Supplement: Attachment — Submitted filename: Response to reviewer.docx [file pone.0326408.s006.docx]

Dear reviewer and editor,

Thank you very much for the thorough review of our submitted manuscript. We are grateful for the detailed comments provided by the reviewer. We have done our utmost to address these comments and would like to submit the revised manuscript.

Below, we have included our responses to the comments received from the reviewer.

For further details, please refer to the "Revised Article with Changes Highlighted" file.

Comment 1

Given the fact that the introduced NOMO-1 cell line is not yet approved by respective regulators or introduced to the MAT context, the authors are strongly advised to start with detailed phenotyping of the used cell line with respect to monocyte/macrophage markers, maturity, and functional analysis in comparison to proper standard primary monocytes. This should be the first critical analysis that can determine further the suitability of the line for the MAT assay.

**Response to Comment 1**

**We acknowledge this suggestion regarding additional data acquisition for phenotyping. We conducted a comparative analysis of TLR expression and CD14 expression (as a monocyte marker) in NOMO-1 cells and PBMCs using qPCR. The new data has been included as Fig 1, and the results and discussion have been incorporated in the manuscript on p10, lines 216-231, and p18, lines 398-408.**

Comment 2

While the assessment of TLR expression is a critical factor to allow for the cell line inclusion in the MAT context, nevertheless the performed and demonstrated comparison of the NOMO-1 TLR expression profile with respect to MM6 or THP1 cells is not a useful or an informative comparison. As the illustrated reference lines (MM6/THP1) are highly debatable and doubted for their suitability in the MAT, with the fact that THP1 cells already failed the MAT validation studies, and MM6 validation status is incomplete. Hence, the TLR expression analysis needs to be compared to and evaluated against primary monocytes expression. In this line, a functional comparison to established MAT cell lines will add more reliable data than comparing only the expression levels.
**Response to Comment 2**

**As we mentioned in our response to comment 1, we obtained data on PBMC for TLR expression analysis and added data for comparison with NOMO-1. Consequently, we have deleted the comparison data with THP-1 and MM6 (removed sFig 1, Fig 5).**

**On the other hand, previous studies have reported the detection of pyrogens using MM6 and THP-1 cells transduced with luciferase reporter genes. These studies were added in the introduction (p4, lines 88-90).**

Comment 3

The authors need to be more accurate in their introduction concerning properly defining NEPs, and the approved cell sources for MAT that also included whole blood MAT. **Response to Comment 3**

**We have added and revised the information about NEPs and cell sources for MAT in the introduction (about NEPs: p3, lines 54-55, about cell sources for MAT: pp3-4, lines 69-73).**

Comment 4

The value of the data needs to be better highlighted throughout the manuscript by conducting the indicated statistical analysis. This is particularly needed when it comes to evaluating the LOD, the authors need to show whether the observed reading at the indicated LOD is of significant difference compared to the untreated control reading.

**Response to Comment 4**

**Regarding the measurements of the LOD, we conducted statistical tests to compare the observed readings of RLU at the indicated LOD with those of the untreated control sample (pp11-12, lines 261-263 and Table 1).**

Comment 5

The authors need to better highlight (perhaps in a separate bar chart) the observed background/noise reading/luminescence at the chosen incubation period at different LPS concentrations. It is a bit confusing why the authors didn’t choose the incubation period of the highest S/N ratio for their assays?

**Response to Comment 5**

**We understand that the points you raised are crucial aspects of the testing conditions. This study was conducted with the goal of establishing a simple and rapid protocol for the MAT. Within this context, we set a 3-hour incubation period as the optimal time to ensure sufficient reactivity. While it is true that longer incubation times can result in increased luminescence values, the results for detecting endotoxins and NEPs at the 3-hour reaction point were consistent with those obtained from longer incubation periods, within the calibration curve range.**

Comment 6

While the authors highlighted in their methods a section for cryopreservation, they didn’t show any data for successful freezing/thawing of functional reporter cells. Such data can add valuable input, to further support the use of the chosen cancer cell line. For example, data for consistency and viability and functionality post thawing is needed.

**Response to Comment 6**

**We apologize for not adequately explaining the testing methods. All of the reporter assay data presented in this paper were obtained using cells immediately after thawing from cryopreserved vials without pre-culture (p8, lines 175-180). Therefore, we believe that we have demonstrated the functionality and consistency of the cells as you suggested.**

Comment 7

In their method part the authors described two versions of virus production with nearly the same wording. At that point it is not clear whether and where two different viruses (lentivirus and undefined virus) were used. If not my mistake, it is strongly recommended to not use the exact wording for both descriptions.
**Response to Comment 7**

**Thank you for your comment. It is our mistake. There was a draft sentence that remained in the manuscript. Only one manufacturing method was used. We have deleted pp6-7, lines 136-146.**

Comment 8

Given the affiliation of the authors and the commercialization interest of Fuji Film in the space of stem cells, the authors should state clearly any potential conflict of interest and whether the work presented will/is planed to be commercialized.

**Response to Comment 8**

**Thank you for pointing this out. We did not consider discussing the commercialization aspects within the manuscript.**

**I would like to ask the editor's advice on where in the manuscript we should address the comments mentioned.**

Other changes

**We have removed the "Data not shown" sections to comply with the PLOS ONE submission guidelines (p11, lines239-240). The reactivity of cells without introduced reporter genes is not a critical point in this paper, so we have deleted the corresponding portions.**

Best regards,

Tomohisa Nanao.
