# Supplementary material for: NOMO-1 cells expressing an NF-κB luciferase reporter gene facilitate a simple, rapid monocyte activation test that can detect a wide range of pyrogens
Source: PLoS One. 2025 Jun 20;20(6):e0326408. doi: 10.1371/journal.pone.0326408 (PMC12180646; doi:10.1371/journal.pone.0326408)
Supplement: Attachment — Submitted filename: Response_to_reviewer_auresp_2.docx [file pone.0326408.s007.docx]

Dear reviewer and editor,

Thank you very much for the thorough review of our submitted manuscript. We are grateful for the detailed comments provided by the reviewer. We have done our utmost to address these comments and would like to submit the revised manuscript.

We received comments regarding language revisions, so we carried out extensive text corrections, including the title. While the text has changed significantly, our arguments and data interpretations remain consistent with our previous submission.

Below, we have included our responses to the comments received from the reviewer.

For further details, please refer to the "Revised Article with Changes Highlighted" file.

Reviewer’s Comment

It is important that the authors clarify whether their results are based on four technical or biological replicates. Biological replicates would include at least four different cell batches that were thawed and seeded, while technical replicates would mean that one frozen cell batch was distributed to four wells for simultaneous measurement. At this point, the present description does not allow an evaluation of whether the NOMO-1 cell line is a reliable and reproducible source for the MAT. Given the importance of the assay and the regulatory requirements it is of utmost importance to demonstrate reproducibility and standardization.

**Response to Comment**

**In many of our experiments, each condition within a single experiment is examined in quadruple as technical replicates, and we conduct three independent experiments using separate cell vials as biological replicates.** **However, it is true that the description of the above conditions in the text was insufficient, making the reproducibility and standardization difficult to understand, and for this, we sincerely apologize.**

**In this revision, we have added descriptions for the technical replicates and biological replicates for each data set. Additionally, in our previous submission, Fig 2 and table 1 only included data obtained from one vial, but we have now added data and included biological replicates from three vials.**

Reviewer’s Comment 1– It should be noted that normalizing TLR or CD14 expression to β-actin levels is not an adequate approach to generally compare and draw conclusions about expression levels between different cell types, which may express β-actin to very different degrees. The authors could give information on the number of replicates for this assay to emphasize their conclusions.

**Response to Comment 1**

**The most important aspect of our qPCR analysis is confirming that TLRs and CD14 are expressed in NOMO-1 cells. However, as you pointed out, increasing the number of replicates is important to enhance the reliability of the conclusions. Therefore, we have added experiments using different cell vials.**

Reviewer’s Comment 2– The authors referred to other established monocytic cell lines that included a reporter-based approach and stated that they performed a more thorough validation with more different TLR targets compared to them. However, a more functional comparison would have been informative, considering the availability of THP1 and MM6 cells in their first draft.

**Response to Comment 2**

**Thank you for your valuable comment. On the other hand, please understand that comparing the currently used NOMO-1 cells, which have undergone further cloning, with the cells that transiently expressed the reporter gene used during the cell line selection is difficult.**

Reviewer’s Comment 3– The authors should include more up-to-date information regarding the European Pharmacopeia. They are no longer just committed to replacing RPT, but have officially announced in July 2024 that they will abolish the RPT.

**Response to Comment 3**

**Thank you for your comment. We have added details about the July 2024 press release to the introduction.**

Reviewer’s Comment 4– The authors display financial disclosures only for TO, although TN, YM, KS, TM and TN are also assigned to FUJIFILM Corporation.

**Response to Comment 4**

**Thank you for your comment. We have added information regarding employment relationships to the cover letter.**

Other comments

Line 4 – pyrogens should be used as plural

Line 22 – pyrogens compromise endotoxins and non-endotoxins, as also later described

Line 23 – pyrogens do not necessarily enter immune cells to induce fever and endotoxic shock

32 – although it became clear later, “NF-kB reporter gene introduced cells” is hard to understand

345 – The detectability “of” NEPs

**Response to Comment**

**We have made the revisions. Please note that we have also made corrections throughout the entire document, so kindly review them.**

Best regards,

Tomohisa Nanao.
