# Supplementary material for: NOMO-1 cells expressing an NF-κB luciferase reporter gene facilitate a simple, rapid monocyte activation test that can detect a wide range of pyrogens
Source: PLoS One. 2025 Jun 20;20(6):e0326408. doi: 10.1371/journal.pone.0326408 (PMC12180646; doi:10.1371/journal.pone.0326408)
Supplement: Attachment — Submitted filename: Response_to_reviewer_auresp_3.docx [file pone.0326408.s008.docx]

Dear reviewer and editor,

Thank you very much for the thorough review of our submitted manuscript. We are grateful for the detailed comments provided by the reviewer. We have done our utmost to address these comments and would like to submit the revised manuscript.

In this review, there were no comments about the manuscript itself. Therefore, no changes have been made to the manuscript. Instead, we received a remark regarding the financial relationship for this scientific work. In response, we have requested the administrative office to update it by the following content in the cover letter.

Reviewer’s Comment

Reviewer #1: The authors have addressed most of the comments. I still ask to disclose any finical relationship for this scientific work.

It is not sufficient to disclose this in the cover letter nor I can see a financial disclose given to the PLOS one editorial office. The authors just say "yes", however a disclosure statement is requested and missing.

**Response to Comment**

**Thank you for your comment. Regarding the financial relationship, we have requested the administrative office to update it by the following content in the cover letter.**

**In the Funding details section:**

**"This study was supported by FUJIFILM Corporation.”**

**In the Competing Interests section:**

**"T.N., Y.M., K.S., T.M., T.K., and T.N. are employed by FUJIFILM Corporation. T.M. and Takahiro Nishibu are also employed by FUJIFILM Wako Pure Chemical Corporation, a group company of FUJIFILM Corporation."**

**"T.O. is affiliated with Hoshi University School of Pharmacy and Pharmaceutical Sciences, which had a collaborative research agreement with FUJIFILM Corporation during the study period."**

**"The funders had no role in study design, data collection and analysis, or preparation of the manuscript."**

**“This study was conducted for the purpose of developing the LumiMAT™ Pyrogen Detection Kit (FUJIFILM Wako Pure Chemical Corporation).”**

Best regards,

Tomohisa Nanao.
